# Burnout and anxiety among Chinese nurses: the mediating roles of positive coping strategies and interpersonal relationship problems: a cross-sectional study

**DOI:** 10.3389/fpsyt.2025.1595392

**Published:** 2025-06-13

**Authors:** Ya Wang, Nan Li, Xia Huang, Jingjun Wang, Junqiang Huang, Yalin Huang, Yan Feng, Liu Qin, Hao Huang

**Affiliations:** ^1^ Department of Nursing, West China Hospital, Sichuan University, Chengdu, Sichuan, China; ^2^ West China School of Nursing, Sichuan University, Chengdu, Sichuan, China; ^3^ West China Tianfu Hospital, Sichuan University, Chengdu, Sichuan, China; ^4^ Mental Health Center, West China Hospital, Sichuan University, Chengdu, Sichuan, China; ^5^ West China Hospital, Sichuan University, Chengdu, Sichuan, China; ^6^ Department of Psychiatry, Dekang Hospital, Chengdu Sichuan, China; ^7^ Cancer Department V, Longhua Hospital Shanghai University of Traditional Chinese Medicine, Shanghai, China; ^8^ Nanchong Hospital Affiliated to Beijing Anzhen Hospital, Capital Medical University, Nanchong, Sichuan, China

**Keywords:** burnout, anxiety, positive coping, interpersonal relationship problems, nurse

## Abstract

**Background:**

Burnout among nurses is a global problem that results in increased turnover as well as decreased career satisfaction and poor nursing service. Previous researchers have suggested that anxiety is associated with burnout. However, the relationship between anxiety and burnout requires further investigation to clarify. How interpersonal relationships and coping styles influence this relationship remains to be further explored. Furthermore, the question of how the demographic characteristics of nurses predict burnout remains unanswered.

**Aims:**

This study aimed to explore the mechanisms by which anxiety affects burnout, to verify the mediating roles of interpersonal relationship problems and positive coping in the relationship between anxiety and burnout, and to explore the factors that can predict burnout among nurses.

**Methods:**

A total of 4,856 nurses were enrolled in this study. The ability of anxiety, positive coping, interpersonal relationship problems, and demographic factors to predict burnout were explored via linear regression models. The relationships among anxiety, positive coping, interpersonal relationship problems, and burnout were also explored by developing a parallel mediation model with the assistance of SPSS PROCESS 3.3 software.

**Results:**

The following factors can predict burnout among nurses: internal medicine ward (β=0.075 p<0.01), surgery ward (β=0.054 p<0.01), operating room (β=0.022 p=0.037), a number of night shifts worked per month higher than 10 (β=0.046 p<0.01), and possession of a master’s degree or higher level of education (β=0.03 p<0.01). Positive coping (β=0.029, 95% CI: 0.022 to 0.036) and interpersonal relationship problems (β=0.134, 95% CI: 0.118 to 0.151) mediate the relationship between anxiety and burnout.

**Conclusion:**

The results of this study reveal that nurses’ department, level of education, and number of night shifts worked per month are effective predictors of burnout. Positive coping and interpersonal relationships problems mediate the relationship between anxiety and burnout.

## Introduction

The concept of burnout was first introduced in the literature in 1974, this issue is viewed as the result of chronic job stress (1, 2). According to the World Health Organization (WHO), burnout is a syndrome that is characterized by three dimensions: 1) feelings of energy depletion or exhaustion; 2) increased psychological distance from work or negative or cynical work-related feelings; and 3) a lack of accomplishment. In the health care industry, nurses are constantly exposed to a stressful work environment and face tremendous workloads. This special work environment has caused nurses to become a high-risk group with regard to burnout, and the World Health Organization (WHO) in particular has noted that the problem of burnout is especially prominent in occupations that require many interpersonal interactions (3).

According to relevant statistics, approximately 11.23% of nurses worldwide have experienced burnout (4). In China, the number of nurses who have experienced burnout is also increasing. A national survey of 51,406 registered nurses in China revealed that half of these nurses had experienced burnout (5). Furthermore, burnout is an important reason why healthcare workers leave their jobs (6). A study of surgical nurses revealed that these nurses’ job satisfaction was affected by burnout. Specifically, as nurses’ levels of emotional exhaustion decrease, their levels of job satisfaction increase (7). In addition, studies have reported that nurse burnout increases the risks of hospitalization and prolonged hospitalization (8). Therefore, alleviating burnout among nurses is an important way of improving nurses’ professional satisfaction and quality of life (9) as well as enhancing the quality of nursing services and patient satisfaction (10). Therefore, an in-depth study of burnout among nurses and its influencing factors is particularly important.

Many demographic factors, such as number of night shifts worked, sleep quality (11), and level of education (12), are believed to be related to burnout. Meanwhile, both clinical experience and previous literature suggest that coping strategies and interpersonal relationships may impact burnout. Effective coping strategies may mitigate the impact of job burnout, while interpersonal conflicts could potentially exacerbate burnout symptoms. In addition, anxiety has been identified as an important cause of burnout among nurses (13, 14), which refers to a common psychological disorder that is characterized by persistent, excessive worry and fear, which are emotions that often impact a sufferer’s daily life negatively. Yu Q et al. reported that the prevalence of anxiety among Chinese nurses was 34% (15). The results of a survey of nurses in several countries revealed that nurses face higher levels of stress and are more likely to experience issues related to mental health, such as anxiety and burnout (16). Both burnout and anxiety are among the most common mental health problems impacting nurses (17). Furthermore, these issues are recognized as important factors that affect nurses’ personal well-being and job performance as well as the quality of patient care and the healthcare system as a whole (15). Coping strategies, interpersonal relationships problems, anxiety and burnout among the nurse population have received a great deal of attention.

## Background

### The relationship between anxiety and burnout

The fact that anxiety leads to burnout has been widely verified (18). Studies have reported that increased anxiety can be viewed as a predictive factor with regard to workplace burnout syndrome. Individuals suffering from anxiety are 2.4 times more likely to experience burnout than are nonanxious people (19). Tan et al. investigated burnout levels among 3,075 Singaporean healthcare workers and suggested that nurses obtained the highest mean burnout scores; furthermore, as their anxiety scores increased, burnout similarly increased (20). The results of a cross-sectional survey of 784 nurses in China also revealed that anxiety is one of the factors influencing emotional exhaustion and depersonalization, which in turn lead to burnout (21). This association reveals that the anxiety status of medical staff may directly impact their levels of burnout, thereby emphasizing the importance of paying attention to and actively managing anxiety and burnout among nurses. Previous studies have reported that individuals’ burnout is associated with anxiety; however, few studies have explored the internal mechanisms by which anxiety affects burnout. Further research on burnout and anxiety should thus be conducted.

### The relationships between burnout and both coping and interpersonal relationships

Moreover, personal coping styles have been reported to be significantly associated with burnout. Spaan et al. revealed that problem-focused coping can help staff mitigate the effects of burnout (22). A cross-sectional study of 385 teachers revealed that effective coping strategies, such as problem solving, exercise and hobbies, improve emotional well-being, whereas negative coping methods lead to psychological distress and reduced work ethic (23). This result is consistent with the findings reported Calegari JG et al., who indicated that negative coping decreases professional quality of life (including compassion satisfaction, burnout, and secondary traumatic stress), whereas positive coping improves such quality of life (24).

In addition, the adverse impacts of the workplace, which result in problems with interpersonal relationships, have been reported to be associated with increased burnout and lower professional fulfillment (25). Generally, nurses’ interpersonal relationships are categorized into interpersonal relationships among healthcare professionals and interpersonal relationships between nurses and their families. The results of a cross-sectional survey of 1,817 nurses in 228 nursing units revealed that the frequent exposure of staff to unsympathetic interpersonal relationships is strongly associated with separation (26). The results of a study of physicians in Bangladesh also demonstrated that physicians who face potential interpersonal conflicts with colleagues or family members are more likely to experience burnout (27).

### The potential mediating roles of coping strategies and interpersonal relationships in the association between burnout and anxiety among nurses

Several studies have reported a link between anxiety and interpersonal relationships, and the relationship between anxiety and coping styles has also been verified (28, 29). These studies have suggested that more positive coping styles are negatively associated with anxiety (30) and that interpersonal relationship problems are associated with anxiety (31). Therefore, it is reasonable to assume that anxiety can decrease a person’s ability to solve problems, thus preventing individuals from adopting more positive coping styles; furthermore, as anxiety levels increase, such individuals abandon assertive and optimistic approaches in favor of helplessness/self-blame and conformity (32). In addition, anxiety reduces the quality of an individual’s interactions with others, thus leading to strained or distant relationships. Prolonged exposure to this situation can cause an individual’s psychological resources to be steadily depleted, which may ultimately lead to burnout.

While, a significant amount of research has explored the associations among anxiety, interpersonal relationship problems and positive coping with burnout, and anxiety, interpersonal relationship problems, and positive coping have been well-established as factors associated with burnout. However, current literature lacks systematic investigations examining both their interrelationships and the concurrent mediating roles of interpersonal relationship problems and positive coping within a unified theoretical framework.

The stress and coping model was proposed by Lazarus. He claimed that the stress responses caused by specific stressors are mediated by two important psychological processes, namely, cognitive evaluation and coping. Stress responses occur when the stressor stimulus exceeds an individual’s coping capacity and perceived resources (33). Therefore, this study aimed to investigate the levels of burnout exhibited by Chinese nurses and to explore the relevant factors that lead to burnout among nurses. Moreover, a stress and coping model was used to guide the present research. Anxiety has been viewed as a source of stress, and burnout has been viewed as a stress response. Interpersonal relationships and positive coping mediate this process. The following hypotheses were proposed in this study:

H1: Anxiety directly affects burnout.
H2: Interpersonal relationship problems mediate the relationship between anxiety and burnout.
H3: Positive coping mediates the relationship between anxiety and burnout.

**Figure 1:**
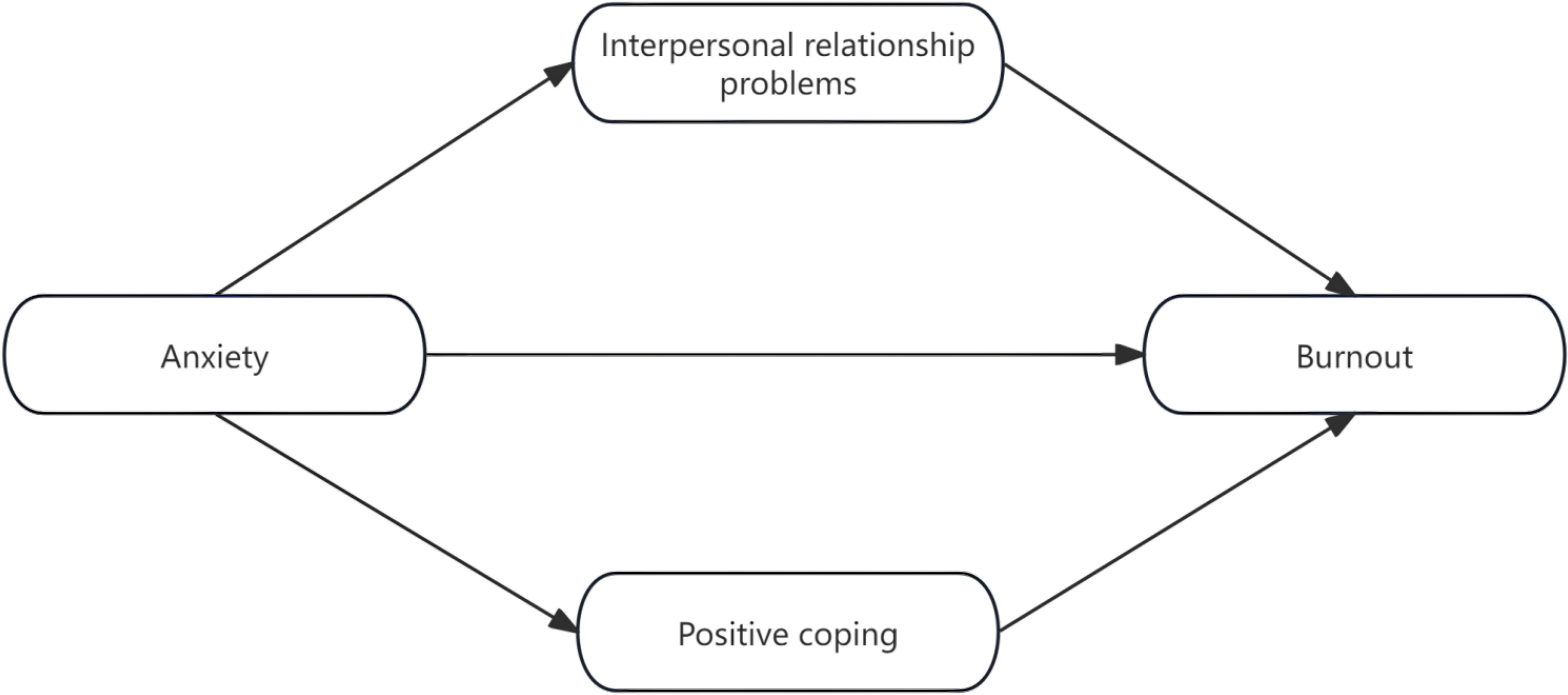
Concept model of this study.

The conceptual model of this study is shown in **Figure 1**.

### The relationships between burnout and other demographic factors

In addition, many studies have indicated that burnout among nurses is caused by several factors, such as monthly income (34). A meta-analytic study of nurses indicated that age is also a significant predictor of burnout (35). Gender has also been shown to be related to burnout; specifically, male nurses are more likely to experience burnout (36). Excessive education and training are also associated with burnout among healthcare workers (6). In addition, certain studies have reported that the work environment is an important factor with regard to burnout among nurses (37); for example, departments such as obstetrics, pediatrics, and emergency medicine have been identified as important environmental factors pertaining to burnout among nurses (38–40).

However, research on the demographic factors that contributes to burnout remains insufficient. For example, previous studies have investigated mostly the effects of night shift length and frequency on burnout, but the specific number of night shifts that are most likely to lead to burnout requires further exploration (41). In addition, the results of studies on the impact of increased levels of education on burnout have been controversial (42–44). Therefore, the purpose of this study was to explore the demographic factors that predict burnout among nurses. The following hypothesis is thus proposed:

H4: Some demographic factors predict burnout among nurses.

## Method

### Sample and settings

This study employed a cross-sectional survey to investigate the related factors influencing nurse burnout. The convenient sampling method was adopted, and the researchers selected study subjects on the basis of specific criteria. The hospitals chosen for this research were public institutions featuring a level III or higher designation (i.e., they contained more than 500 beds), community hospitals, and private hospitals. The inclusion criteria were as follows: (1) aged 18 years or older;(2) ability to understand the questionnaire; and (3) status as a registered nurse. Participants were excluded if they met any of the following conditions: (1) diagnosed cognitive impairment or severe mental health disorders; (2) on medical or personal leave for >1 month during the survey period. In the demographic information section of the questionnaire, participants were required to provide their personal information. The investigators screened the questionnaire and excluded subjects who did not meet the inclusion criteria. Statistical significance was accepted as p<0.05. for all analyses.

### Investigation procedures

An online questionnaire was created with the assistance of WJX software (www.wjx.com). This questionnaire included three components: the study’s purpose and method, an informed consent form, and a formal questionnaire. These questionnaires were distributed online. After obtaining consent from head nurses working in 10 hospitals, the online questionnaire was sent to the head nurse of each department. The precautions taken in this research and the topics under investigation were explained to the head nurse of each department by the researchers and were further communicated to nurses from head nurses. Subsequently, the questionnaires were sent to nurses via WECHAT software and were completed by nurses within a week. Only after the nurses read the instructions for at least 30 seconds and signed the informed consent form were they allowed to complete the questionnaire.

**Figure 2:**
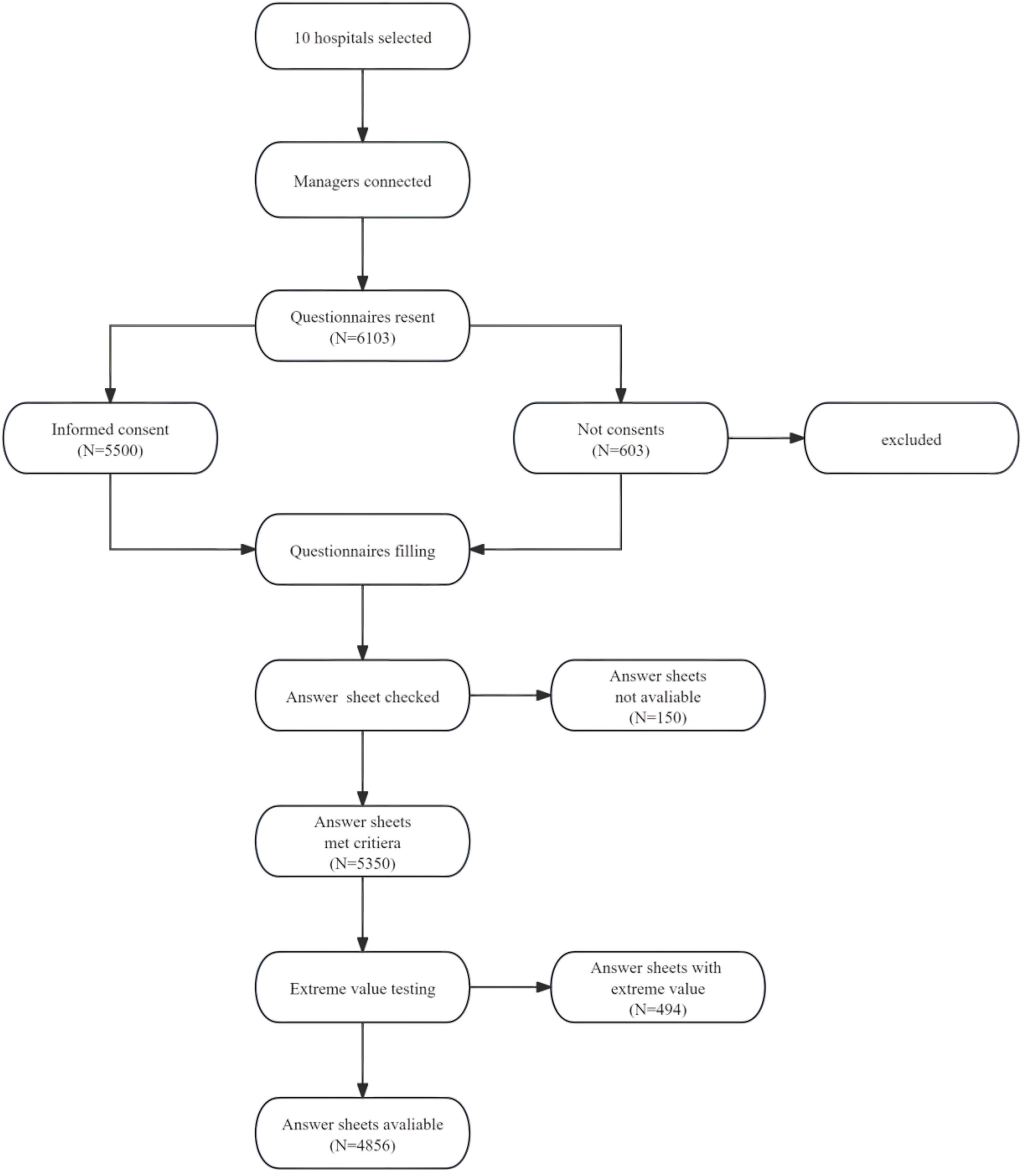
The process of sampling.

From March 2022 to September 2022, 6,103 online questionnaires were distributed, and 5,500 nurses were selected to participate in this research, to ensure sufficient sample size and geographic diversity. After the screening process, 5,350 nurses met the inclusion criteria. This study implemented rigorous quality control measures to ensure the authenticity of the questionnaire data. The criteria employed in these measures included (1) a requirement that the questionnaire be completed in more than 1 minute; (2) the exclusion of questionnaires featuring identical answers; (3) the exclusion of incomplete questionnaires and (4) each participant completed the questionnaire only once, and no longitudinal interventions or repeated measurements were conducted. Questionnaires were included in this study only if they fully met these conditions, and after extreme efforts with regard to value testing and data cleaning, 4,856 questionnaires were included in the study. The details of the sampling process are demonstrated in **Figure 2**.

## Measures

### Demographic characteristic questionnaires

The participants completed demographic characteristic questionnaires that collected their gender, age, level of education, professional title, marital status, only child status, fertility status, department, clinical experience, monthly number of night shifts worked, hospital level, position, fatigue due to nurse–patient relationships, and willingness to attend psychological training courses provided by the hospital.

### Anxiety

The Depression-Anxiety-Stress Self-Rating Scale-Brief Version (DASS-21) was used to assess respondents’ anxiety. This scale was revised by Xu et al. in 2010 to include 7 items, and it uses a 4-level scoring system ranging from “0” (very inconsistent) to “3” (very consistent). The total scores were obtained by multiplying the sum of all the items by 2; the higher the score is, the more anxious the participant in question. The reliability analysis indicated a Cronbach’s α coefficient of 0.858 for this scale.

### Burnout

The Maslach Burnout Inventory-General Survey (MBI-GS), which was developed by Schaufeli et al., contains three dimensions, i.e., emotional exhaustion, depersonalization, and decreased personal accomplishment, that contain 16 items (45). The Chinese version of the MBI-GS was revised by Chaoping Li et al. and contains 15 items (46); this version features a 7-level scoring system ranging from “0” (never occurring) to “6” (occurring daily). The total burnout score was standardized by first calculating the mean of all 15 items and then multiplying by 20 to convert it to a 0–120 scale, facilitating comparison with prior studies using similar normalization methods. Final scores of 50, 75, and 100 on this scale represent the thresholds for mild, moderate, and severe burnout, respectively, and scores below 50 are considered to indicate the nonexistence of burnout. A higher total score indicates a greater degree of job burnout among the participants in this research. The Cronbach’s α coefficient for the Chinese version of this inventory was 0.959.

### Interpersonal relationships

The scale used to measure interpersonal relationships was developed by Deng Ruichang and was used to assess the degree of interpersonal relationship problems experienced by respondents (47). The interpersonal relationships scale consists of 28 items across four dimensions, which assess the degree of distress experienced by respondents in the contexts of talking, socializing, dealing with others and socializing with members of the opposite gender; a score of 1 point was assigned if the respondent met the criteria described by the entries, while a score of 0 points it was assigned otherwise. The higher the total score on this scale is, the greater the degree of interpersonal relationship distress reported by the participants in this study. The Cronbach’s α coefficient for this scale was 0.914.

### Positive coping styles

This study used the Simple Coping Scale (SCSQ) developed by Yaning Xie, which includes 20 items; items 1 through 12 measure characteristics pertaining to positive coping, while items 13 through 20 measure characteristics pertaining to negative coping (48). In this study, only the positive coping style scale, which is scored on a 4-point scale ranging from “0” (not taken) to “3” (often taken), was used to assess the degree to which respondents coped positively with negative events; in this context, the higher the score obtained by a respondent is, the more positive that respondent’s coping style. The Cronbach’s α coefficient of the scale was 0.920, thus indicating good reliability.

### Statistical analysis

Data analysis was conducted with the assistance of SPSS 26.0 software. Frequency and percentage statistics were used to describe the categorical variables. Normality of continuous variables was assessed using the Kolmogorov-Smirnov test supplemented with skewness and kurtosis values. The quantitative data that follow a normal distribution are presented in terms of the means ± standard deviations (SDs), while non-normally distributed quantitative data are described as median (Inter quartile Range). Since the normality assumption of burnout scores was violated, non-parametric tests were performed to examine differences in burnout among nurses with different demographic characteristics. Spearman’s correlation analysis was employed to explore the relationships between burnout and continuous variables such as anxiety. A multiple linear regression analysis included factors with significant differences as independent variables. The independent variable factors that affect the dependent variable were identified through multiple linear regression analysis. Parallel mediation analysis was conducted via Model 4 of the SPSS PROCESS 3.3 macro with the goal of testing the mediating roles of positive coping and interpersonal relationship problems in the relationship between anxiety and burnout, and variables were standardized (z-scores) to generate standardized regression coefficients (β) for mediation analysis. This transformation does not affect statistical significance but facilitates interpretation of effect sizes in SD units. Demographic variables (education level, night shifts) were included as covariates to control for potential confounding but excluded from the mediation diagram to focus on core psychological constructs. Parallel mediation analysis was prioritized to evaluate the hypothesized mediation effects, whereas multiple linear regression was used to explore the predictive effects of factors such as demographic characteristics on burnout. These methods are complementary and address distinct research questions. The chi-square test was used to determine whether any significant differences were present within the burnout population in terms of their participation in psychological training programs or the ways in which they dealt with the nurse–patient relationship.

## Results

### Demographic features of the subjects

A total of 4856 nurses were included in this study; these nurses were predominantly female (4706, 96.9%), and the median age of participants was 30 years (IQR: 26-35). Most of these nurses had obtained a bachelor’s degree or higher level of education (3218, 66.2%), among whom 16 (0.3%) had obtained a master’s degree or higher level of education. The departments in which these nurses worked mainly included internal medicine wards (1535, 31.5%), surgery wards (1085, 22.3%), and operating rooms (256, 5.3%). The median duration of nursing clinical experience was 9 years (IQR: 5-13), although fewer nurses worked more than 10 night shifts per month (414, 8.5%). More than half of the 4,856 nurses (2707, 55.7%) who participated in this study felt fatigued due to the nurse–patient relationship, and 3,904 (80.4%) believed that hospitals should provide psychological programs; further details are presented in **Table 1**.

**Table 1:** Differences in burnout among nurses by Socio-demographic characteristic of participants (N = 4856).

Variables	N (%)	Median (IQR)	H (Z)	p
Gender			-.2.241	0.025
Female	4706 (96.9)	29.33 (17.33-41.33)		
Male	150 (3.1)	24.00 (13.33-40.00)		
Department			41.356	<0.001
Internal medicine ward	1535 (31.5)	30.67 (20.00-44.00)		
Surgery ward	1085 (22.3)	30.67 (18.67-41.33)		
Outpatient	222 (4.6)	28.00 (18.67-38.67)		
Emergency room	252 (5.2)	26.67 (13.33-42.67)		
Operating room	256 (5.3)	30.00 (18.67-40.00)		
Gynecology	292 (6)	26.67 (14.67-40.00)		
Pediatrics	230 (4.7)	28.00 (16.00-40.00)		
Stomatology	20 (0.4)	28.00 (11.00-38.67)		
Psychiatry	70 (1.4)	27.33 (12.00-41.33)		
ICU	141 (2.9)	29.33 (17.33-41.33)		
Others	753 (15.5)	28.00 (17.33-38.67)		
Hospital level			4.068	0.397
Grade II B	250 (5.1)	32.00 (17.33-44.33)		
Grade II A	877 (18.1)	30.67 (18.67-41.33)		
Grade III B	1284 (26.4)	28.00 (17.33-40.00)		
Grade III A	2427 (49.8)	29.33 (17.33-41.33)		
Others	18 (0.4)	30.67 (18.33-37.33)		
Professional title			2.373	0.499
Primary	3143 (64.7)	29.33 (17.33-41.33)		
Middle	1389 (28.6)	29.33 (18.67-41.33)		
Associate senior	294 (6.1)	29.33 (18.67-40.00)		
Senior	30 (0.6)	29.33 (13.00-36.33)		
Marital status			1.887	0.596
Unmarried	1303 (26.8)	29.33 (18.67-41.33)		
Married	3406 (70.1)	29.33 (17.33-41.33)		
Divorces	138 (2.8)	26.67 (16.00-41.33)		
Others	9 (0.2)	25.33 (16.67-32.67)		
Only child or not			-1.272	0.203
Yes	1609 (33.1)	29.33 (17.33-42.67)		
No	3247 (66.9)	29.33 (17.33-40.00)		
Education level			19.836	<0.001
Polytechnic school	96 (2.0)	20.67 (9.67-33.33)		
Junior college	1542 (31.8)	29.33 (17.33-41.33)		
Bachelor degree	3202 (65.9)	29.33 (18.67-41.33)		
Master degree and above	16 (0.3)	29.33 (24.33-52.00)		
Fertility status			2.831	0.418
Childless	1728 (35.6)	30.67 (18.67-41.33)		
1 Child	2144 (44.2)	29.33 (17.33-40.00)		
2 Children	968 (19.9)	29.33 (17.33-41.33)		
3 Children or above	16 (0.3)	28.67 (12.00-43.00)		
Monthly night shift			89.076	<0.001
0	1506 (31)	26.67 (16.00-38.67)		
1—2	359 (7.4)	30.67 (20.00-41.33)		
3—4	700 (14.4)	28.00 (16.00-40.00)		
5—6	604 (12.4)	29.33 (17.33-41.00)		
7—8	812 (16.7)	29.33 (18.67-41.33)		
9—10	414 (8.5)	32.00 (18.67-44.00)		
>10	461 (9.5)	36.00 (23.33-49.33)		
Positions			7.204	0.206
Standardized training students	12 (0.2)	26.67 (19.33-42.33)		
Nurses	4025 (82.9)	29.33 (17.33-41.33)		
Nursing team leaders	348 (7.2)	29.33 (18.67-41.33)		
Head nurses	394 (8.1)	32.00 (21.00-41.67)		
Department head nurses	55 (1.1)	30.67 (14.67-37.33)		
Superintendent of nursing department	22 (0.5)	27.33 (14.00-51.58)		

^a.^ p-values derived from Mann-Whitney U (binary), Kruskal-Wallis H (multi-category).

^b.^ IQR, Inter quartile Range.

### Comparison of levels of burnout

To investigate differences in burnout among nurses with different demographic characteristics, non-parametric tests were prioritized due to the non-normal distribution of burnout scores, which were standardized (z-scores) for comparability across analyses. Significant differences in burnout were observed among nurses with different genders, departments, levels of education, and average number of night shifts worked. See **Table 1** for further details.

### Correlations among the major variables

All continuous variables were standardized using z-score transformation. And spearman’s correlation analysis was conducted since the continuous variables were nonnormally distributed. The results revealed that the anxiety experienced by nurses was closely related to their levels of burnout (r=0.646 p<0.01). Positive coping styles were negatively related to nurses’ burnout (r=-0.343 p<0.01). In contrast, poor interpersonal relationships were positively associated with nurses’ burnout (r=0.557 p<0.01). Furthermore, anxiety, positive coping styles, and interpersonal relationships were revealed to be related to each other. A stepwise forward multiple linear regression model was constructed based on the results of the correlation analysis. See **Table 2** for further details.

**Table 2:** The correlations among the continuous variables by Spearman’s Correlation (N=4856).

Variables	Skewness (SE)	Kurtosis (SE)	Median (IQR)	1	2	3	4	5	6
Age	1.057 (0.035)	0.707 (0.070)	30.000(26.000-35.000)	1					
Clinical experience	1.198 (0.035)	1.090 (0.070)	9.000(5.000-13.000)	0.943^**^	1				
Anxiety	1.320 (0.035)	1.936 (0.070)	6.000(2.000-12.000)	0.015	0.035^*^	1			
burnout	0.924 (0.035)	1.452 (0.070)	29.333(17.333-41.333)	-0.015	-0.001	0.646^**^	1		
Interpersonal relationship problems	0.978 (0.035)	0.278 (0.070)	5.000(1.000-10.000)	0.001	0.016	0.576^**^	0.557^**^	1	
Positive Coping	-0.013 (0.035)	-0.674 (0.070)	24.000(17.000-30.000)	0.059^**^	0.047^**^	-0.325^**^	-0.343^**^	-0.315^**^	1

^a.^ Bold values indicate statistically significant associations (*p < 0.05, **p<0.01).

^b.^ SD, standard deviation; SE, standard error; IQR, Interquartile Range.

^c.^ Absolute skewness >1.96 or kurtosis >1.96 may indicate substantial non-normality. All variables met acceptable thresholds, supporting the use of non-parametric analyses as described in Methods.

^d.^ Non-normally distributed variables are summarized as median (IQR); normally distributed variables are reported as mean ± SD.

### Multiple linear regression

The level of burnout was used as the dependent variable, and the factors associated with burnout were included as independent variables with the goal of constructing multiple linear regression equations. Independent variables included in the stepwise multiple linear regression were selected based on significant differential associations with burnout (p < 0.05) from demographic comparisons (**Table 1**) and Spearman’s correlation (**Table 2**). Categorical variables (e.g., educational level) were dummy-coded with reference category as the baseline, while continuous variables (anxiety, positive coping) were standardized using z-score transformation. The results revealed that internal medicine ward (β=0.161, 95% CI: 0.114 to 0.209), surgery ward (β=0.131, 95% CI: 0.078 to 0.183), operating room (β=0.098, 95% CI: 0.006 to 0.191), average number of night shifts worked per month>10 (β=0.158, 95% CI: 0.089 to 0.227), master’s degree or higher level of education (β=0.520, 95% CI: 0.171 to 0.869), anxiety (β=0.486, 95% CI: 0.462 to 0.511), and interpersonal relationship problems (β=0.244, 95% CI: 0.220 to 0.268) represent risk factors with regard to increased burnout; furthermore, positive coping (β=-0.095, 95% CI: -0.117 to -0.074) was revealed as a protective factor that could decrease burnout. See **Table 3** for further details.

**Table 3:** Multivariate linear regression analysis of burnout as the dependent variable.

Variables	B	β	t	p-Value	LLCI	ULCI
Department (REF:Others)						
Internal medicine ward	0.161	0.075	6.654	<0.001	0.114	0.209
Surgery ward	0.131	0.054	4.864	<0.001	0.078	0.183
Operating room	0.098	0.022	2.082	0.037	0.006	0.191
Monthly night shift (REF:0)						
>10	0.158	0.046	4.506	<0.001	0.089	0.227
Education level (REF: Polytechnic school)						
Master degree and above	0.520	0.030	2.920	0.004	0.171	0.869
**Anxiety**	0.486	0.486	39.151	<0.001	0.462	0.511
**Interpersonal relationship problems**	0.244	0.244	19.586	<0.001	0.220	0.268
**Positive Coping**	-0.095	-0.095	-8.759	<0.001	-0.117	-0.074

^a.^ REF, reference. ^b.^ Bolded terms denote major variable categories (e.g., Education Level, Department). ^c.^ Non-bolded terms represent specific subgroups within each category that significantly differ from the reference group (e.g., Polytechnic school, Others).

### Mediation analysis based on parallel mediation analysis

A parallel mediating analysis that included demographic factors as control variables and interpersonal relationship problems and positive coping as mediating variables was conducted. The results of the moderated mediation analysis revealed that the total effect of anxiety on burnout was estimated to be β=0.649 (95% CI: 0.628 to 0.671). The direct effect of anxiety on burnout was estimated to be β=0.486 (95% CI: 0.462 to 0.511). The total indirect effect of anxiety on burnout was estimated to be β=0.163 (95% CI: 0.145 to 0.180), and the total indirect effect accounted for 25.12% of the total variance in burnout. Furthermore, the mediation analysis indicated that interpersonal relationship problems mediate the relationship between anxiety and burnout (β=0.134, 95% CI: 0.118 to 0.151) and that positive coping mediates the relationship between anxiety and burnout (β=0.029, 95% CI: 0.022 to 0.036). All reported mediation effects (indirect/direct) are standardized coefficients (β) derived from z-transformed variables. Details regarding the mediation analysis are presented in **Table 4**.

**Table 4:** Parallel mediation analysis results: indirect effects of anxiety on burnout through interpersonal relationship problems and positive coping.

Variables	Total effect			Direct effect			Indirect effect		
	B	LLCI	ULCI	B	LLCI	ULCI	B	LLCI	ULCI
Anxiety	0.649**	0.628	0.671	0.486**	0.462	0.511	0.163**	0.145	0.180
Interpersonal relationship problems(M1)							0.134**	0.118	0.151
Positive coping(M2)							0.029**	0.022	0.036
Difference of indirect between M1 to M2							0.106**	0.087	0.125

^a.^ Bold values indicate statistically significant associations (p**<0.01).

^b.^ Bootstrapped 95% confidence intervals (5,000 resamples).

## Discussion

**Figure 3:**
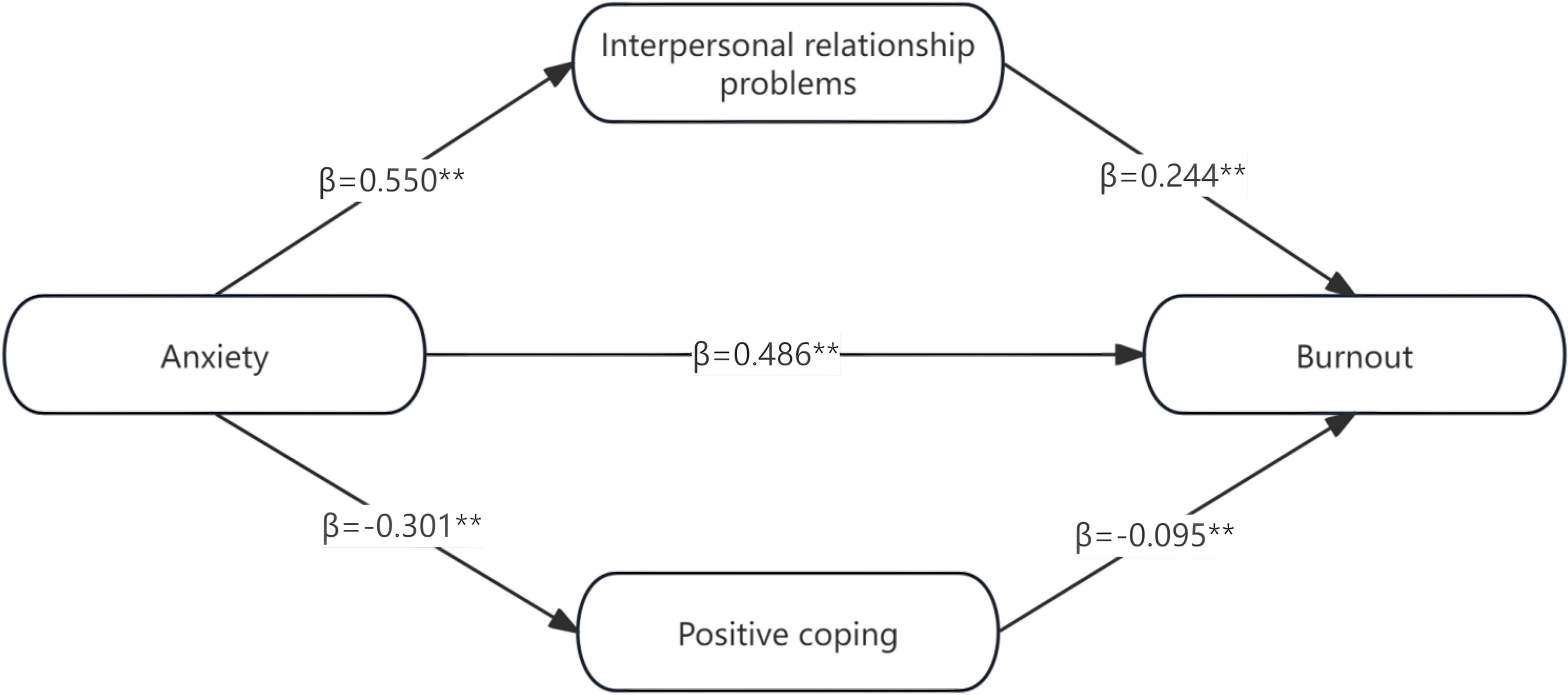
Mediation model of this study. **indicates statistical significance at the p<0.01 level.

The purpose of this study was to investigate the factors that predict burnout with the goal of determining whether positive coping and relationship problems mediate the relationship between anxiety and burnout. The results of this in-depth study reveal a range of demographic factors that are linked to burnout. Specifically, nurses working in medical wards, surgical wards, and operating rooms, those who work more than ten night shifts per month and those who possess a master’s degree or higher level of education are more likely to experience burnout. These findings are consistent with H4. In addition, nurses who face the challenges of anxiety and interpersonal distress are more likely to be impacted by burnout, and positive coping can help alleviate such burnout. In addition, positive coping and relationship problems are revealed to mediate the relationship between anxiety and burnout. These findings are in line with H1, H2 and H3. The research model is illustrated in **Figure 3**.

### Night shift

This study demonstrates that the number of night shifts worked per month is a predictor of burnout. Specifically, working more than ten night shifts is revealed to be a risk factor for burnout among nurses. Previous studies have reported that rotating night shifts can damage the physical and psychological health of nurses (49). In terms of psychological status, night shifts can lead to depression, anxiety and occupational stress among nurses, and these emotional problems are important causes of burnout (50). In terms of physiological health, night shifts cause nurses to be more susceptible to sleep disorders and cardiovascular diseases, and these physiological factors can aggravate burnout (51). A study of the perceived professional benefits reported by Chinese nurses also revealed that prolonged night shifts can lead to low sleep quality scores and decreased job satisfaction, which in turn lead to low levels of perceived professional benefits (52). A study of 1,774 Chinese nurses conducted by Liao T et al. also revealed that good sleep quality helps alleviate emotional exhaustion (53). In summary, it is reasonable to assume that nurses who work more than 10 night shifts per month are more likely to suffer from anxiety and other forms of emotional distress as well as from physical discomfort, which may be accompanied by disrupted sleep patterns; these impacts can exacerbate burnout among nurses.

However, night shifts are essential to ensure that patients receive proper care overnight. Therefore, it is necessary to find a balance that can minimize this negative impact while ensuring the continuity of care for patients. This study reveals that the frequency of monthly night shifts can predict burnout among nurses, thereby providing support for clinical departments’ efforts to rationalize the number of monthly night shifts worked by nurses. Specifically, by adjusting the number of night shifts worked by nurses per month, we can address their physical health and psychological needs in a more humane way.

### Level of education

Several studies on burnout among Chinese nurses have reported that a nurse’s level of education affects burnout (5, 54, 55). One study revealed that nurses’ level of education is significantly associated with the depersonalization dimension of burnout (56). A systematic evaluation and meta-analysis of practitioners in the oncology profession revealed a significant difference between nurses with a low level of education and nurses with a high level of education in terms of the dimension of depersonalization (57). The results of the study conducted by Siam BGAH et al. revealed that nursing staff who have obtained only a low level of education face relatively high levels of burnout (58). Zhang et al. reported that nurses who have obtained higher levels of education and work experience tend to be assigned more challenging jobs, thus leading to higher levels of personal fulfillment and higher incomes (5).

However, the results of this study reveal that a master’s degree or higher is a significant predictor of burnout among nurses, which is not consistent with the findings of previous research. To explain this phenomenon, we speculate that, among the 16 nurses included in this study who had obtained master’s degrees or higher levels of education, 11 were younger than 35 years old, only 3 had a monthly income greater than 10,000 RMB, 9 had obtained only primary professional titles, 9 were first-line charge nurses, and more than half were required to work night shifts. In summary, these young nurses with master’s degrees or higher levels of education had not obtained gained higher incomes or more challenging work due to this high level of education. Moreover, to complete their studies or advance in their careers, nurses with master’s degrees or higher levels of education must make responsibility for more academic missions, and to complete this aspect of such academic missions, they require more rest time. In this case, they face higher levels of occupational pressure and burnout than do nurses with lower levels of education. In conclusion, we must pay more attention to young nurses with master’s degrees or higher levels of education to determine whether they are experiencing burnout and require additional psychological support.

### Anxiety has a direct effect on burnout

The results of a cross-sectional survey of 2,705 healthcare professionals reveal that more than half of the healthcare professionals included in this survey exhibit varying degrees of anxiety (59); furthermore, these healthcare professionals exhibit significantly higher levels of anxiety than do members of the general population (17). A large stream of research has reported that anxiety leads to burnout (60). Liao T et al. noted that anxiety is significantly associated with the emotional exhaustion dimension of burnout, a condition which may be caused by the poor working environments faced by nurses and the excessively high workloads they face (53). Griffin BJ et al. concluded that persistent anxiety or increased anxiety can lead to lower job satisfaction and job engagement, which in turn lead to increased burnout and willingness to leave one’s job (61).

This finding is consistent with the hypothesis proposed in this study, namely, that anxiety directly affects burnout. It is reasonable to claim that persistent anxiety consumes a great deal of mental energy, thus causing individuals to feel tired and powerless at work. In addition, anxiety may interfere with an individual’s concentration and reduce the individual’s work efficiency, thereby exacerbating work stress and leading to the emergence of a vicious cycle resulting in burnout. Therefore, implementing effective measures to help nurses alleviate their anxiety is an important way of improving their job satisfaction and alleviating burnout.

### The parallel mediating roles of positive coping and interpersonal relationship problems

The results of a survey of 322 health professionals reveal that coping strategies contribute significantly contribute to the levels of health anxiety exhibited by health professionals and that the coping strategy of self-blame contributes significantly to anxiety (62). In addition, some studies have reported that both positive attitudes and positive coping styles can significantly predict burnout and improve the quality of nurses’ professional lives (24). Babore A et al. reported that positive attitudes are the strongest protective factor against distress and that the higher the individual’ level of positive attitudes is, the lower the level of distress that this individual experiences; furthermore, positive attitudes are the best strategy with regard to relieving occupational stress (63). Zakaria N et al. reported that dysfunctional coping is related to poor mental health and that dysfunctional coping leads to a significant increase in the scores pertaining to three dimensions of burnout (49).

In this study, positive coping is revealed to mediate the relationship between anxiety and burnout, which is consistent with the hypotheses proposed in this study. Nurses who experience lower levels of anxiety tend to employ more effective coping mechanisms. In addition, nurses who employ more positive coping strategies are less susceptible to emotional exhaustion and exhibit lower burnout. These findings are similar to those reported by Jiao R et al., who indicated that positive coping mediates the relationship between workplace bullying and nurse burnout, that nurses’ quality of life can be significantly improved through positive coping styles, and that positive coping styles can reduce nurse burnout (64). Therefore, for nurses with anxiety, the adoption of positive attitudes and coping styles, such as problem solving, exercise and hobbies, can help alleviate personal anxiety and reduce burnout.

Moreover, anxiety, as an emotional disorder, has been reported to be predictive of interpersonal relationships. A meta-analysis of longitudinal studies featuring 115,133 participants was conducted to assess the relationships between social anxiety and the quality levels of four social relationships (including family-related, school-related, romantic, and general relationships). The results of that study revealed that social anxiety negatively predicts the quality of social relationships (65). This finding is consistent with the results of the present study, which indicate that anxiety is positively related to interpersonal relationship problems. Moreover, previous researchers have shown that relational coordination among employees is positively correlated with increased employee career satisfaction and decreased burnout (66). Larsman P et al. also reported that interpersonal relationship distress leads to conflicts related to ethical values, which in turn result in burnout (26), and a study of school employees revealed that reduced social connection leads to burnout (67).

Our study reveals that interpersonal relationship distress mediates the relationship between anxiety and burnout, in line with our expectations. First, anxiety may cause individuals to feel nervous and uneasy in social situations, which may adversely affect their communication skills and social interactions. Second, anxiety may affect individuals’ self-esteem and self-confidence. Such anxiety may cause them to experience low self-esteem and insecurity during interpersonal interactions, thus leading them to be excessively cautious or concerned about their behavior and speech in social situations, which may in turn affect their interactions with others. Third, anxiety may cause individuals to exhibit excessive dependence in the context of relationships. The corresponding inability to assert their own needs may cause these individuals to experience greater stress within their interpersonal relationships. Thus, anxiety leads to increased levels of interpersonal relationship problems, and interpersonal relationship problems lead to more severe burnout, which is consistent with the findings presented above.

This study establishes through structural equation modeling that positive coping and interpersonal relationship problems as parallel mediators between anxiety and burnout, elucidating an underexplored mechanistic pathway. These findings providing new insights into how anxiety translates into burnout through these mechanisms. Thus, to alleviate nurse burnout, measures such as psychological training programs are necessary. Researchers have suggested that psychological interventions should be provided to nurses at an early stage by their organizations, which can help nurses prepare for possible psychological stressors and social stressors in advance (49). The results of an 8-week intervention study revealed that psychological training can effectively influence nurses’ well-being and alleviate both burnout and occupational stress (68). However, the results of this study revealed that nurses experiencing burnout are more likely to feel fatigued as a result of the nurse–patient relationship. These interpersonal relationship problems are also more likely to aggravate nurses’ burnout, thus leading to a vicious cycle. Moreover, we reveal that nurses experiencing burnout are reluctant to attend psychological training. We speculate that these attitudes may be related to the excessive willingness of nurses experiencing burnout to leave their jobs or to the resistance of such nurses to the possibility of participating in psychological courses at institutions. Therefore, understanding nurses’ willingness to participate in psychological training and the implementation of measures aimed at encouraging active participation are important ways of helping nurses alleviate burnout. In addition, most psychological training requires nurses to participate during their personal time, and the question of whether this situation impacts these nurses’ rest time, thereby increasing burnout among these nurses, requires further exploration.

### Strengths and limitations

By constructing structural equation models, this study is able to validate the risk factors that can predict burnout among nurses, to identify the mediating roles of positive coping styles and interpersonal relationship problems in the relationship between anxiety and burnout, and to provide a theoretical basis for predicting burnout among nurses. In addition, the willingness of nurses experiencing burnout to participate in psychological training is explored based on a comparison, revealing that such nurses are more likely to experience fatigue due to the nurse–patient relationship and more reluctant to participate in psychological training. These findings highlight the necessity of providing psychological training to nurses experiencing burnout.

This study has several limitations. (1) This study employs a cross-sectional design, and does not clearly reveal how factors such as department, level of education, number of night shifts worked monthly, anxiety, interpersonal relationship problems, and positive coping affect nurses’ burnout over time, which requires further research. (2) This study explores only the mediating role of positive coping styles in this context, and the impact of negative coping on burnout requires further exploration in the future. (3) This study focuses only on nurses who have obtained licenses to practice in some provinces and cities in China and does not obtain a complete sample from each province.

## Conclusion

This study reveals that working in internal medicine wards, surgery wards, or operating rooms, an average number of night shifts worked per month higher than 10, and possession of a master’s degree or higher level of education are all risk factors for the occurrence of burnout, and it verifies that positive coping and interpersonal relationship distress play mediating roles in the relationship between anxiety and burnout. Therefore, it is particularly important to provide psychological training programs for nurses experiencing burnout, and providing such nurses with more psychological support can help them improve their interpersonal relationships and encourage them to adopt more positive coping styles. This approach can help alleviate burnout and improve nurses’ career satisfaction.

## Data Availability

The original contributions presented in the study are included in the article/supplementary material. Further inquiries can be directed to the corresponding author.
